# State Aid for Needy Mothers*An address delivered before the State Conference of Charities and Correction at Fort Worth on December 1, 1913, by Dr. Theo. Y. Hull, of San Antonio, Secretary State Society of Social Hygiene.

**Published:** 1914-02

**Authors:** 


### Department of Eugenics.

Conducted by Dr. Malone Duggan and Dr. Theo. Y. Hull.

### THE TEXAS STATE SOCIETY OF SOCIAL HYGIENE.

Headquarters: San Antonio, Texas.

DR. MALONE DUGGAN, President, San Antonio.
DR. F. E. DANIEL, 1st Vice President, Austin.
PROF. A. CASWELL ELLIS, 2d Vice President, Austin.
MRS. ELI HERTZBERG, 3d Vice President, San Antonio.
DR. THEO. Y. HULL, Secretary, San Antonio.
MR. W. L. EVANS, Treasurer, San Antonio.

EXECUTIVE COMMITTEE.

Dr. Malone Duggan, Chm.
Dr. Theo. Y. Hull, Sec'y.
Dr. W. F. McCaleb.
Hon. C. H. Jenkins.
Rev. A. W. S. Garden.
Hon. J. C. Willacy.
Mr. W. L. Evans.
Dr. Frank D. Boyd.
Dr. J. R. Nichols.
Dr. J. M. McCutchan.
Dr. W. A. King.
Dr. J. W. Torbett.
Dr. F. C. Walsh.
Dr. Joe Reuss.
Dr. Frank Paschal

For Texas Medical Journal.

### State Aid for Needy Mothers.*

The proposition to compensate the mother for the constructive part she assumes in the making of the State, while not altogether new to the world, has found recent application in this country. The idea that has dominated the discussion in the Old World is entirely separate and distinct from that which is uppermost in the New. For some time the Eastern nations have been facing the problem of a decreasing birth rate. France, which for a generation has been studying ineffectively the proposition, is now face to face with the fact that her deaths annually exceed her births by many thousands. England, though to a less degree affected, is watching a steady decline in her birth rate. Australia, with a large area of sparsely populated territory, is offering inducements to parenthood. Germany, whose people have long been noted for their virility, suddenly finds herself confronted with a rapid decline in births, which soon threatens to bring her population to a standstill. Even in this country, the decline is noticeable, though largely counteracted by the constant influx of new blood from other parts of the world.

The solution to this problem is sought in the East by offering inducements for parenthood. Mohammed, the prophet of Islam, long ago sought to encourage the growth of large families by offering a free pass to Paradise to all fathers of ten children. And

*An address delivered before the State Conference of Charities and Correction at Fort Worth on December 1, 1913, by Dr. Theo. Y. Hull, of San Antonio, Secretary State Society of Social Hygiene.

probably earlier than this the Roman Empire tried to discourage celibacy and childlessness by various bounties and penalties on bachelors and the heads of families of only one child. More recently the Australians have devised a scheme to encourage parenthood by granting a maternity allowance of $25 for every child born in the Commonwealth, and has further proposed to pension all mothers at the age of 45 years having six living children. The English insurance bill provides a maternity allowance of $7.50 for the wife of an insured workman.

In this country the idea of encouraging parenthood has scarcely entered the field of discussion, but another and far different idea has prevailed. In this, the State acknowledges that it has a duty to the children of certain classes. The laws enacted in the various States so far distinctly aim to provide home training and home surroundings for the children of widowed and needy mothers. In doing this, the State does not seek to compensate the mother for the part she has already borne, but seeks to employ her in an attempt to lessen the State's burden of inefficiency, delinquency and crime. Constantine, when in 315 A. D. he decreed that assistance from the public treasury should be given to parents too poor to support their children, had no such advanced conception of the duty of the State. His idea, though commendable, was merely to prevent infanticide and the selling of children into slavery by such parents. This was a common practice, happily now unknown, but the same tendency still exists, and finds expression in child labor.

The State long ago conceived the duty to provide for the education of all the children in its jurisdiction. It has been very liberal in taxing the people for this purpose. It has, in many instances, assumed the duty to compel those who fail to take advantage of the local educational facilities to do so. It does this in the belief that an educated citizen is much more valuable to the State than the ignorant citizen. It believes that general enlightenment is essential to republican government. The conception of the State's duty embodied in the various mothers' pension acts is still more advanced. This legislation embodies the idea that a considerable proportion of delinquency is due to the lack of proper home training. Since such delinquency often leads to crime and, therefore, burdens the State, it becomes a matter of wise government to provide a plan, when poverty is the cause, whereby the evil may be corrected or prevented. The State expects to realize on its investment an increased efficiency in that portion of its citizenship which through poverty might be led into crime.

The idea finding expression in these various legislative acts is to provide for the needy at a time when such aid will be productive of the most good both to the child and to the State. The granting of an allowance to the widowed and needy mother with children to support rests upon the basis of service rendered to the State. We have long been familiar with the idea of granting pensions to soldiers or their widows and orphans for services rendered the State in time of need, as in war. The idea of pensioning the needy mothers is a recognition that the work of the mother in rearing children is a work for the State in the same sense that bearing arms in defense of one's country is a work for the State. Many a mother in her efforts to train her little ones into good citizens has suffered more severely than the wounds of battle.

The various enactments are an acknowledgment that the mother is the proper one to rear and train her own child. They go further than this. They recognize the mother's right in her child, providing she is a suitable person for its care. They also recognize the fact that the State has an interest in the protection of the child, the family and the home. Their very enactment is an acknowledgment that the protection of the home is in the interest of public morals and the prevention of poverty and crime.

The government is interested in the number and character of its citizens. The higher the moral tone, the smaller is the labor of governing. If children are neglected because of poverty, and thereby the tendency to worthlessness, dependency and crime is increased, the task of governing is correspondingly greater and the State suffers proportionately. If the natural mothers in their homes can rear better children and train them to become more efficient citizens, the State profits by their labor. In a sense, the mother, by the bearing of children, makes the State. On her falls the heavier burdens of society, and society is, therefore, under obligations to the mother to see that her lot is not unnecessarily hard. It is a just compensation, a law of nature, that they who bear the burden of reproduction must be protected.

The aim of the State, as organized society, is to perpetuate itself under the most favorable conditions. We may look upon this as the business of the government. When we ask that widowed and needy mothers be granted an allowance whereby they may care for their children, we may ask it as a business proposition. The State already makes an allowance for education. It recognizes the training of the children, as in the provisions for their instruction at public expense, as a part of the State's business. In this country, it is considered the soundest of business principles. The State's duty is to preserve, protect and instruct the child-life, for the children become the future citizens and represent the future life of the State.

In our National Congress, in our State Legislatures, and even in our municipal councils, there is constantly heard the demand for economy. Yet the same bodies are continually appropriating public funds for building and maintaining institutions that represent waste. Prisons and reformatories and asylums and almshouses represent waste, not alone in wealth but in human life. They do not train human beings into better and more efficient citizens. They simply impound and maintain the vicious and more unfortunate elements of human society. The home, on the other hand, represents a true economy, since it preserves the child-life and trains it for efficient citizenship.

The government has for decades assumed the right to enact tariff laws for the dual purposes of revenue and protection. It has assumed that certain industries necessary to the growth of the country must be supported. It has assumed the right to levy an indirect tax for their support. The greatest industry of the country is the rearing and training of its children, that they may become competent citizens. It is admitted that there are many weak points in this industry. The granting of allowances to needy mothers should be looked upon in the same light—a support and not a charity. It is an obligation. The rearing of children is the mother's business. It is a business of the first importance to the State. If we have a right to tax the people for the support of weak industries, some of which are of doubtful utility to the State, we certainly have a similar right to levy a tax for the support and upbuilding of weak families, for we know to a moral certainty that these weak families, if not built up, will become charges upon the revenues of the State.

Since the home, the family unit, is the basis of our government, since it is the foundation of society and the unit of civilization, its protection becomes the first and most important duty of society. It is the simple duty of the State to insure the sacredness of the home and the proper protection of the child. The granting to the needy mother an allowance on which to rear her child or children is just. It is just to the whole people, for they share in its benefits.

The children of parents unable to support them are, under present conditions, placed in institutions supported by the various charity organizations or by the State. It has very often been questioned whether the conditions obtaining in these institutions are all that may be desired. We have had no Charles Dickens to turn on the sidelights. Our own natural instincts tell us that the child was intended to be brought up in a home and by a mother. No stretch of the imagination can make us believe that it was designed to be brought up en masse, as must be the case in any institution. Food, clothing and shelter do not meet the demands of childhood. Even though the ultimate object may be to adopt the child into some family, the real home conditions are rarely attained. It is the mother that makes the real home. The institution cannot be a real home to the waif, and the family into which it may be adopted rarely becomes such, for the real mother is not there to listen to the childish tale of woe and to counsel wisely.

If our real purpose be to help the child, we must pay some attention to the effects of this homeless training upon the child's after-life. Most competent observers believe that the conditions and surroundings in an institution blight the child for life. Something either comes into the child-life that were better omitted or something is taken out of the child-life that should remain. Be this as it may, a study of the children admitted into the Elmira Reformatory shows that 60 per cent were brought up in institutions for dependent children. It is well known that the graduates of reformatories make doubtful citizens. There is something wanting in the training of such children, and that something, in the vast majority of the cases, is the mother. Institutional training rarely develops the innate good in the child. The home plan, which this legislation implies, is expected to reduce this 60 per cent very materially by helping to develop the good qualities of the child, rather than the bad, by the presence of the love and the guidance of the mother. Had this plan been adopted twenty years ago by the city of Chicago, how much would the mother love have reduced that city's annual bill for crime? The city now pays $600,000.

As unfavorable as the mental and moral effects of institutional training must be upon the child, there is still another lesson that the State may profit by. Taking the question of the cost to the

State into consideration, the difference is very much in favor of the home. George Creel says that a child may be kept in the home for one-third what it costs in an institution. This is probably somewhat of an exaggeration. The records of Massachusetts for October, 1912, show that 1613 children were provided for in their homes at an average pension expenditure of $6.89. The Chicago Juvenile Court cared for 327 needy mothers with 1200 children in their homes at an average cost of $5.75 per month per child. The institutional plan costs $10 per month per child, for the same period.

An instance or two may illustrate this phase of the case more clearly. Two boys, whose want of home training was due to the fact that their mother was compelled to work away from home to provide them with necessary food, clothing and shelter, found their way into a reform school, where they cost the State $15.16 each per month. The mother, on less than two-thirds of what it cost the municipality, could have remained in her home and have made good citizens out of them. This would have represented a wise economy and good business, for it would have fitted them for honorable lives rather than that of public charges. In a second instance, a destitute mother at the death of her husband was compelled to place her seven children in an asylum. This cost the county at least $60 per month. Later, when the Mothers' Allowance Law went into effect, the children were returned to the mother, who was granted an allowance of $22 per month for their support. How much more the allowance saved the community by reason of the better citizenship attained by these seven waifs the future alone can tell. It would seem a wise policy, and far cheaper, to train good citizens than to prosecute criminals, made so by the dual conditions, poverty and neglect.

Before entering upon a detailed discussion of the essential features of these enactments, we might with profit note the rapid growth of this form of legislation. In 1908, the city of San Francisco put in operation the first work of this kind. In 1911, Kansas City entered the list of American cities that sought to provide for the children of needy mothers. On April 7th of that year the Governor of Missouri signed the first law granting a pension to needy mothers with dependent children. This act was amended in 1913, and according to the Secretary of State for Missouri the law is operative only in Jackson county. The Illinois Legislature passed a similar law, but this, like the Missouri law, was applied only in Chicago. The new Illinois law, approved June 30, 1913, applies to the several counties in the State. The Colorado Legislature enacted a Mothers' Compensation Law in 1912. Tc June 16, 1913, Mothers' Pension Laws have been enacted in the following States, viz.: California, Colorado, Idaho, Illinois, Iowa, Massachusetts, Michigan, Minnesota, Missouri, Nebraska, New Jersey, Ohio, Oregon, Pennsylvania, South Dakota, Utah and Washington. Wisconsin later adopted similar legislation. (Eighteen States in all). This is one of the most remarkable showings in the history of legislation, when we consider the brief period that the subject has been before the public, and is one of the most gratifying evidences of the progress of the child welfare movement in this country. The law as enacted by the State of Illinois and approved June 30, 1913, is entitled "An Act to provide for the partial support of mothers whose husbands are dead or have become permanently incapacitated for work by reason of physical or mental infirmity, when such mothers have children under fourteen years of age, and are citizens of the United States of America and residents of the county in which application for relief is made. And also to provide for the probationary visitation, care and supervision of the family for whose benefit such support is provided." The title of this act quite clearly defines its scope. The essential features of the law are those relating to (1) its administration, (2) the party to whom an allowance may be made, (3) the conditions upon which relief may be granted, (4) the age of the child or children for whom relief is sought, (5) the amount of the allowance, and (6) probation officers and their pay.

Section 1 of the Illinois statute provides that the juvenile court, or where there is no such court, the county court shall have original jurisdiction in all cases. The administration of the law varies slightly in the different States. In California, Missouri, Ohio and Washington, it is said to be administered by the juvenile court alone; by the juvenile court or some other county court of competent jurisdiction in Colorado and Oregon; by the juvenile court or the county commissioners in Utah; by the various courts in Idaho, Iowa, Michigan, Minnesota, Nebraska, New Jersey and South Dakota; by the city or town overseers of the poor in Massachusetts, and in Pennsylvania by an unpaid board of five to seven women residents of the county, appointed by the Governor.

Section 2 of the Illinois statute provides that a woman whose husband is dead or permanently incapacitated for work by reason of physical or mental infirmity may file an application for relief. The application of this section of the law is also slightly different in the various States. *Poverty, however, is the one factor underlying all.* It applies to any parent who is unable to properly support a dependent or neglected child, but who is otherwise a proper guardian for it in California, Colorado and Nebraska; to mothers only in Massachusetts, Michigan, Ohio, Pennsylvania and Washington; to mothers whose husbands are prisoners in Idaho, Iowa, Minnesota, Missouri, Ohio, Oregon, South Dakota and Washington; to mothers whose husbands are confined in the State insane asylums in Iowa, Minnesota, Missouri, Oregon and Washington; to mothers whose husbands are incapacitated for labor in Minnesota, Ohio, Oregon, South Dakota and Washington; to divorced and unmarried mothers in Michigan alone, and to an appointed guardian where neither parent is found fit in California and Colorado.

Section 11 of the Illinois laws specifies the conditions upon which the relief may be granted. It provides (1) that the child must be living with its own mother; (2) that it is for the welfare of the child to remain at home with the mother; (3) that the relief shall be granted only when the mother would be required to work regularly away from the home and her child; (4) that the mother must be a proper person, physically, mentally and morally fit to bring up the child; (5) that the relief shall be necessary to prevent neglect of the child; (6) that the mother is poor; (7) that the mother must be a resident of the country for at least three years; and (8) that there are no relatives of sufficient ability to support the child. The laws of the various States predicate relief on practically the same conditions. The Oregon law states the purpose and intent of the act to be to keep the child or children under the guidance and control of the mother.

Section 12 of the Illinois act provides for relief of children up to the age of fourteen years, and in case of any sick or incapacitated child the time may be extended to sixteen years of age. The age limit in the other States varies slightly. It is fourteen years in Iowa, Massachusetts, Missouri and South Dakota; fifteen years in Idaho, Utah and Washington; sixteen years in Nebraska, New Jersey and Oregon; seventeen years in Michigan; the legal working age in Ohio and Pennsylvania; and no limit is set in California and Colorado.

Section 10 of the Illinois law places the maximum allowance at $15 per month where the mother has but one child under fourteen years of age, with a further allowance of not to exceed $10 per month for each remaining child under age limit; provided, that the total amount paid any one mother shall not exceed $50 per month. The Oregon law states that the allowance shall be $10 for one child and $7.50 for each additional child. The allowances vary in the other States all the way from $6.25 per month in California to $15 per month in some States. There is no maximum amount in two or three of the States. Some of the States make an allowance of $5 to $12 per month for additional children. Three States—California, Ohio and South Dakota—limit orders granting such allowances to six months.

Section 14 of the Illinois statute relates to probation officers and their pay. In order to carry out successfully a law of this character, and to prevent fraud, it is necessary that some officer or officers should be appointed to keep close watch over the beneficiaries of the act. This section gives the court the power to appoint one or more qualified persons of good character to serve as such officer or inspector during the pleasure of the court, to be paid a suitable compensation by the county, the amount to be settled by the county board. The Oregon law makes no specific reference to probation officers, but gives the court full power to summons witnesses, compel their attendance and pay them the same as in criminal cases. The Colorado law gives the juvenile in counties of over 100,000 inhabitants and the county court in other counties power to appoint proper persons to investigate, visit, keep records, and make reports in all cases requiring relief. It also contains a provision for limiting the number of such investigators and the amount of salaries to be paid them.

The *source of the funds* needed to carry these laws into effect is the county treasury in every State except Massachusetts. In that State, the city or town treasury supplies the amount needed. In three—California, Massachusetts and Pennsylvania—State aid is granted. In Colorado the law provides another source of income. The law directs that counties of over 20,000 population shall establish and maintain workhouses or facilities for the detention and employment of men who have been convicted of non-support of women and children. Any money collected for the labor of such persons must be used for the maintenance of the fund necessary for the carrying out of the provisions of this act. The payment of the earnings of the fathers convicted of non-support into a fund for the support of such dependent children is intended to place a premium on motherhood in favor of the mother who bears the child.

Another feature of the Colorado law that seems wise is the power given the court to substitute an equivalent in supplies or assistance for the cash allowance. Under this provision the board of county commissioners may provide the home with rent, groceries, clothing, etc., until such time as it may be safe to pay the cash.

The Colorado act takes another step in advance, in that it affirms the parent's right to the child. The owner of a piece of property has always been assumed to have a right therein which could not be taken away even by the government without a reasonable compensation. The mother of a child which she is unable to support is in practice supposed to lose all right to the child when it is taken in charge by the State. The State has held the right to adopt out such children regardless of the parents' wishes, and, even though the parent became able to provide for the child, there was no power of recovery.

Under the old law in Colorado, no legal rights were given to the parents for the reclamation of the child. The law of 1908 provided that when the parents became able to support the child and educate it (if not indentured) the child might be restored to them, but it was not compulsory on the board to do so. In practice, it was found that this provision was of little value, for the children were usually adopted out. The State Home for Dependents was merely intended as a State detention home until such time as the child might be placed in some family. The Mothers' Compensation Law provided that the child might be reclaimed when poverty was shown to be the cause of detention, but this showing must be made at the time of commitment. The intention of the new law was to give the real parents a reasonable chance to recover and provide for the child. The theory of this form of legislation is that many children are separated from their mothers by reason of poverty alone; that these children come into the juvenile court for forms of waywardness or delinquency, due to neglect, and that this neglect was due to the fact that the mother was compelled to go out to earn the support for herself and children. Many cases might be cited to sustain this theory.

The Missouri law was the first to be enacted, and its purpose was to keep the mother in the home with her children. This is for the protection of the child, the home and the State. When children are long neglected, they tend to become delinquent. The poor suffer from physical and moral neglect. Undoubtedly, delinquency unchecked leads to the penitentiary. The first care, therefore, is to bolster up the home. In this respect, there is a general gain. The child gains by not being pauperized. The mother gains in self-respect, because she is the mistress of a home and is doing her natural duty to her child. The citizens gain in the social recognition accorded the mother's services and the child's needs. The State gains, in that it is preventing waywardness, delinquency and crime, and is, therefore, in its effort to assist the needy mother to properly rear her children, serving its own best interests.

